# Synchronous Diagnosis of Squamous Cell Carcinoma of the Lung and Mixed Cellularity Hodgkin Lymphoma of the Nasopharynx

**DOI:** 10.7759/cureus.32885

**Published:** 2022-12-23

**Authors:** Binoy Yohannan, Arthi Sridhar, Johncy J Kachira, Zubaida AlJumaili

**Affiliations:** 1 Hematology and Medical Oncology, University of Texas Health Science Center at Houston, Houston, USA; 2 Health Services Research, MD Anderson Cancer Center, Houston, USA; 3 Pathology and Laboratory Medicine, University of Texas Health Science Center at Houston, Houston, USA

**Keywords:** synchronous tumors, tobacco smoking, synchronous cancers, multiple primary tumors, classic hodgkin's lymphoma, squamous cell lung carcinoma

## Abstract

Hodgkin lymphoma (HL) is a highly curable B cell lymphoproliferative neoplasm with a bimodal age distribution. Lung cancer is the leading cause of cancer-related deaths in both sexes. We present a rare case of synchronous squamous cell carcinoma (SCC) of the lung and mixed cellularity HL of the nasopharynx. A gentleman in his 70s presented with right-sided chest pain and shortness of breath. CT of the chest showed a peripheral lung mass, and a biopsy confirmed SCC of the lung. The patient underwent a positron emission tomography/computed tomography (PET/CT) for staging that revealed an ^18^F-fluorodeoxyglucose (FDG)-avid mass in the nasopharynx. Flexible nasal endoscopy and biopsy of the nasopharyngeal mass revealed mixed cellularity classical HL. The patient was started on chemoimmunotherapy for lung cancer. Unfortunately, two months after initiation of treatment, the patient died from COVID-19 pneumonia and multiorgan failure.

## Introduction

Multiple primary tumors (MPTs) are increasingly being recognized in men older than 50 years, with a reported incidence of around 2%-17% [1,2]. MPTs can be either synchronous or metachronous depending on the timing of diagnosis, with the latter being more common. It is well known that long-term survivors of Hodgkin lymphoma (HL) are at an increased risk of lung cancer, especially those who have received radiotherapy. However, the synchronous presentation of lung cancer and HL is extremely rare, with only two cases published previously [3,4]. We present an interesting case of a synchronous diagnosis of lung cancer and HL of the nasopharynx in an elderly man.

## Case presentation

A gentleman in his 70s with hypertension, hyperlipidemia, diabetes, and abdominal aortic aneurysm treated with endovascular repair presented with a one-month history of dull aching, constant right-sided chest wall pain. He tried non-steroidal anti-inflammatory drugs without any significant relief. He reported significant shortness of breath and difficulty sleeping on the right side due to chest pain. The patient denied fever, chills, cough, palpitation, and hemoptysis. He reported poor appetite and 5 kg weight loss. The patient reported a 50-pack-year smoking history. He was a social drinker but denied illicit drug use. Physical examination was unremarkable except for right chest wall tenderness.

**Figure 1:**
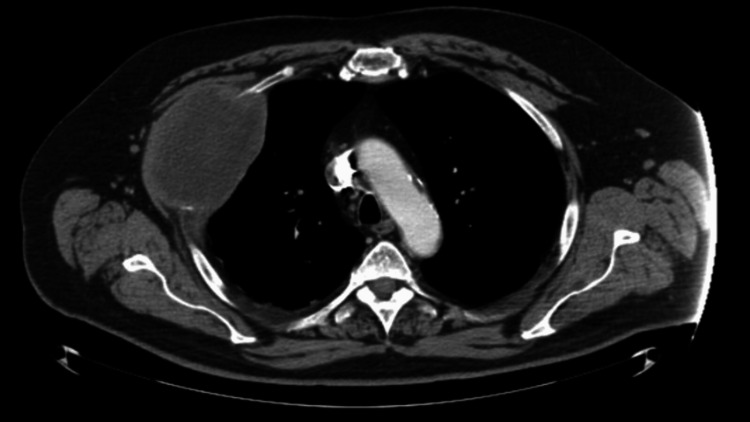
CT of the chest (axial view) showing a well-circumscribed low-attenuation ellipsoid mass measuring approximately 10.3 × 6.6 cm involving the right anterolateral chest wall.

Computed tomography (CT) of the chest showed a large right chest wall mass arising from the right third rib (Figure 1).

**Figure 2:**
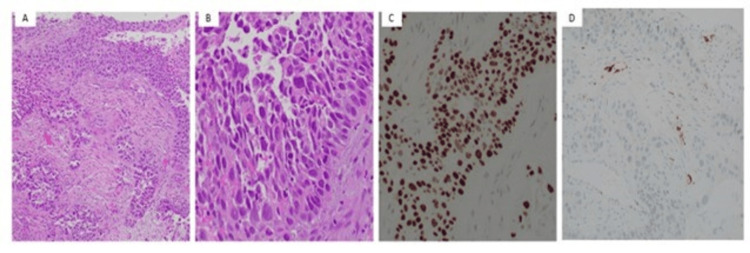
(A, B) (at 40x and 100x): core needle biopsy of the chest wall cystic mass showing invasive moderately differentiated squamous cell carcinoma. (C) IHC P40 is positive for the malignant cells. (D) The malignant cells do not express P16. SCC, squamous cell carcinoma

He was also found to have a pulmonary embolism and was started on systemic anticoagulation with low-molecular-weight heparin. He underwent CT-guided biopsy of the mass, and pathology showed squamous cell carcinoma (SCC) of the lung (Figure 2).

**Figure 3:**
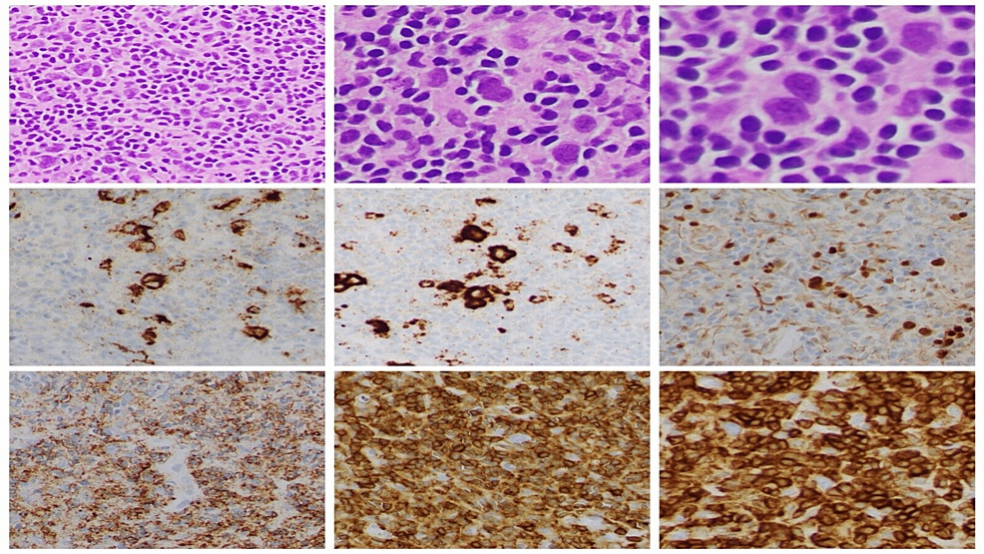
The nasopharynx midline biopsy specimen showing scattered multinucleated HRS cells, in a background of small lymphocytes. The HRS cells show prominent eosinophilic nucleoli (Figures 1A–1C, at 100×, 200×, and 400×, respectively). Some cells are positive for immunohistochemical stains CD15 and CD30 with membranous and Golgi pattern (Figures 1D, 1E). PAX5 stain shows weak intensity for the HRS cells (Figure 1F). CD20, CD45, and CD3 stains, respectively, did not highlight the HRS cells (Figures 1G–1I). HRS, Hodgkin and Reed-Sternberg

CT of the abdomen and pelvis did not show any evidence of metastatic disease. A positron emission tomography/computed tomography (PET/CT) was performed to complete the staging work-up that showed an 18F-fluorodeoxyglucose (FDG)-avid mass in the nasopharynx with a maximum standardized uptake value of 6.2. The nasopharynx is a highly unusual site of metastasis for lung cancer. Therefore, the patient underwent transnasal biopsy; pathology is shown in Figure 3.

An Epstein-Barr encoding region (EBER) in situ hybridization study was positive. The morphological findings, together with immunophenotype results, were most consistent with classic HL, mixed cellularity subtype.

Given his advanced chronic obstructive pulmonary disease and poor lung reserve, he was not a candidate for surgical resection. The patient was started on systemic chemoimmunotherapy with carboplatin, paclitaxel, and pembrolizumab for lung cancer. Two months after the initiation of treatment, he presented with COVID-19 pneumonia and died from multiorgan failure.

## Discussion

MPT is a well-recognized phenomenon. Any anatomic site can be involved; however, the lungs, gastrointestinal tract, and genitourinary tract tend to be more commonly affected [2]. Ares et al. reported that patients with MPT often have a strong family history of cancer [5]. In addition, replication errors in microsatellite loci are also believed to be an important contributing factor [6].

Among MPTs, synchronous presentation of a solid tumor and a hematological malignancy is extremely rare and poses a formidable diagnostic and therapeutic challenge. The exact pathophysiology of synchronous solid tumors and hematological malignancies is unknown; however, aging and immunosuppression are the likely culprits [2]. Lung cancer and HL have a few risk factors in common. Tobacco smoking is the most important risk factor for lung cancer and is an independent risk factor for lymphomas (both Hodgkin and non-Hodgkin), which have a direct correlation with the intensity of smoking [7]. Our patient had an extensive smoking history, which significantly increased his risk for both lung cancer and HL. Epstein-Barr virus (EBV) infection could also be a common pathogenic link. Approximately 30%-50% of HLs and almost all cases of HIV-associated lymphomas harbor EBV, and it is an adverse prognostic marker [8]. A causal link between EBV infection and non-small cell lung cancer is controversial and has not been clearly established. However, in the endemic regions, EBV infection may play a pivotal role in the pathogenesis of certain subsets of lung cancers such as pulmonary lymphoepithelioma-like carcinoma [9-12].

Head and neck lymphomas usually arise from Waldeyer’s ring and can manifest as different histological subtypes, including diffuse large B cell lymphoma and NK/T cell lymphoma. Nasopharyngeal HL is extremely rare, accounting for less than 1% of HL cases. In a retrospective study of 3500 patients with HL, Iyengar et al. reported only nine cases of nasopharyngeal HL [13]. The nasopharynx is believed to be a reservoir for EBV [14]. Our patient was EBER positive, and this likely explains the unusual site of HL.

The diagnosis of synchronous malignancies is challenging. In patients with biopsy-proven malignancy, it is often unnecessary to biopsy every metastatic site. However, there must be a high index of clinical suspicion for a second primary malignancy when there is an abnormal tumor mass or FDG-avid uptake at sites unusual for metastatic disease. Nasopharynx is a highly unusual site for metastatic disease, although there are few case reports of lung cancer (both squamous and adenocarcinoma) with nasopharyngeal metastasis [15,16]. We were concerned about the possibility of a second primary malignancy given his significant smoking history and that prompted the additional work-up that confirmed HL.

A solid tumor synchronous with a hematological malignancy poses a therapeutic challenge, and there is no consensus regarding the optimal treatment approach. MPTs are also a barrier to clinical trial enrollment, as patients with second primary neoplasms are generally excluded. In general, a multidisciplinary approach that considers the stage of each cancer type, aggressiveness of the malignancy, and patient performance status must be considered when selecting a treatment regimen, and the priority should be given to the most aggressive cancer. When possible, the oncologist must choose a regimen that can effectively treat both cancer types and take appropriate steps to mitigate treatment-related toxicity. Immune checkpoint inhibitors (ICIs), either alone or in combination with chemotherapy, have become an important pillar in the management of non-small cell lung cancer. Similarly, ICIs are highly effective in classic HL due to the genomic amplification of chromosome 9p24.1 [17]. There are promising data showing high rates of complete remissions with the use of ICIs in the frontline setting in patients with early-stage HL [18]. Hence ICIs are an effective therapeutic strategy that can treat both lung cancer and HL.

## Conclusions

In summary, this case highlights a rare case of synchronous lung cancer and nasopharyngeal HL. Oncologists should have a high clinical suspicion for a second primary malignancy when tumors are seen in unusual locations outside of the usual metastatic sites.
